# The impact of staging FDG-PET/CT on treatment for stage III NSCLC - an analysis of population-based data from Ontario, Canada

**DOI:** 10.3389/fonc.2023.1210945

**Published:** 2023-08-23

**Authors:** Craig A. Beers, Gregory R. Pond, James R. Wright, Theodoros Tsakiridis, Gordon S. Okawara, Anand Swaminath

**Affiliations:** ^1^ Division of Radiation Oncology, Juravinski Cancer Centre, Hamilton, ON, Canada; ^2^ Faculty of Health Sciences, McMaster University, Hamilton, ON, Canada

**Keywords:** positron emission tomography, PET, non-small cell lung cancer, NSCLC, Ontario, Canada

## Abstract

**Purpose:**

Fluoro-2-deoxyglucose positron-emission tomography (FDG-PET/CT) is now considered a standard investigation for the staging of new cases of stage III NSCLC. However, there is not published level 3 evidence demonstrating the impact of FDG-PET/CT on appropriate therapy in this setting. Using retrospective population-based data, we sought to examine the role and timing that FDG-PET/CT scans play in influencing treatment choice, as well as survival in patients diagnosed with stage III NSCLC.

**Materials and methods:**

A retrospective cohort of patients diagnosed with stage III NSCLC from 2009-2017 in Ontario were identified from the IC/ES (formerly Institute of Clinical Evaluative Sciences) database. FDG-PET/CT utilization over time, trends in mediastinal biopsy technique and usage, the impact of FDG-PET/CT on overall survival (OS), and its influence on use of concurrent chemoradiotherapy (CRT) were explored. The impact of timing of pre-treatment FDG-PET/CT on OS was also analyzed (≤28 days prior to treatment, 29-56 days prior, and >56 days prior).

**Results:**

Between 2007 and 2017, a total of 13 796 people were diagnosed with stage III NSCLC in Ontario. FDG-PET/CT utilization increased over time with 0% of cases in 2007 and 74% in 2017 with pre-treatment FDG-PET/CT scans. The number of patients who received a mediastinal biopsy similarly increased in this timeframe increasing from 41% to 53%. More patients with pre-treatment FDG-PET/CT scans received curative-intent therapy than those who did not: 23% vs 13% for CRT (p<0.001), and 23% vs 10% for surgery (p<0.001). Median OS was longer in those with FDG-PET/CT scans prior to treatment (17 vs 11 months), as was 5-year survival (22% vs 14%, p<0.001), and this held true on both univariate and multivariate analyses. Timing of FDG-PET/CT scan relative to treatment was not associated with differences in OS.

**Conclusion:**

Improvements in OS were seen in this cohort of stage III NSCLC patients who underwent a pre-treatment FDG-PET/CT scan. This can likely be attributed to stage-appropriate therapy due to more complete staging using FDG-PET/CT. This study stresses the importance of complete staging for suspected stage III NSCLC using FDG-PET/CT, and a need for continued advocacy for increased access to FDG-PET/CT scans.

## 1 Introduction

Over the last decade, the diagnosis and staging of non-small cell lung cancer (NSCLC) has significantly improved primarily with the use of fluoro-2-deoxyglucose positron-emission tomography (FDG-PET/CT). Previous randomized controlled trials have demonstrated the utility of FDG-PET/CT (typically in combination with CT) with respect to avoidance of futile surgery in patients with both early stage and more advanced NSCLC (1–3). The current guidelines from the National Comprehensive Cancer Network (NCCN) recommend FDG-PET/CT scans for staging of all newly diagnosed patients with NSCLC, and also as a potential tool to evaluate (4, 5) response to treatment as well as for ongoing surveillance (3).

Within Ontario, Canada, FDG-PET/CT became an insured indication for any new diagnosis of NSCLC within 2 years following a 2007 evidence review suggesting its utility (1). However, the downstream evidence to date that supports PET in terms of influencing staging, stage-appropriate therapy such as chemoradiation (CRT) and surgery for stage III disease, and potentially survival is limited. A subgroup analysis of the randomized PROCLAIM trial reported an improvement in both progression-free and overall survival in patients who had undergone a staging FDG-PET/CT scan, therefore underpinning the importance that FDG-PET/CT scans have as an early investigation for NSCLC (2). At a population-based level, the magnitude of effect of FDG-PET/CT on survival to our knowledge has not been previously investigated. Furthermore, information on the impact of FDG-PET/CT on therapy choice, and timing of FDG-PET/CT relative to curative treatment and outcomes has been limited.

Our objective was to conduct a population-based cohort study over the period of 2007-2017 in order to determine the magnitude of influence of FDG-PET/CT on both management and outcomes in patients with stage III NSCLC. Our hypothesis was that use of pre-treatment PET would result in improved survival as well as increased access to curative therapy such as CRT and surgery. Secondary objectives included the type and utilization of mediastinal biopsies, timing of FDG-PET/CT relative to initiation of any treatment in the entire cohort, as well as the years 2014-2017 using cut off dates of FDG-PET/CT scans ≤28 days prior to treatment start, 29-56 days prior to treatment, and >56 days prior to treatment.

## 2 Methods

### 2.1 Databases

A retrospective, population-based cohort of patients with stage III NSCLC treated in Ontario, Canada, was identified between January 2007 and March 2017. This cohort was obtained using databases contained within IC/ES (formerly known as the Institute of Clinical and Evaluative Sciences). IC/ES provides linked health administrative data from multiple sources on all residents in the province of Ontario. Because most Ontario residents are insured through a universal, single payer health care plan, they would be captured in the IC/ES registries. Databases selected for inclusion from IC/ES included the Ontario Cancer Registry (OCR), which provides diagnosis and staging details, Ontario Health Insurance Plan (OHIP) for physician billing codes to determine visits, and treatment dates, and Cancer Activity Reporting Level (ALR) for details regarding systemic and radiation treatments.

### 2.2 Patients

Patients with NSCLC were selected based on the International Classification of Diseases (ICD) for Oncology morphology codes. ALR codes were used to determine patients treated with curative-intent radiation and chemotherapy. Study ethics approval was obtained through the local institutional Hamilton Integrated Research Ethics Board and the Data Analytics Systems programs through IC/ES.

Data collection for this study used a start date of 2007 as this was the year FDG-PET/CT guidelines for NSCLC were released. Inclusion criteria for the study were a diagnosis of NSCLC based on ICD-10 topography codes C34.x (C.34.1-C34.9) and histology codes for NSCLC (8046/3), carcinoma NOS (8010/3), large cell carcinoma NOS (8012/3), large cell neuroendocrine carcinoma (8013/3), large cell undifferentiated carcinoma (8020/3), anaplastic carcinoma (8021/3), pleomorphic carcinoma (8022/3), papillary squamous cell carcinoma (8052/3), squamous cell carcinoma NOS (8070/3), adenocarcinoma NOS (8240/3), adenocarcinoma mixed subtype (8255/3), papillary adenocarcinoma NOS (8260/3), and bronchioalveolar cell carcinoma (8250/3). Patients also had to have documented stage III disease, no previous diagnoses of cancer ≤ 5 years of NSCLC diagnosis, had not received radiation or chemotherapy treatment prior to NSCLC diagnosis, and not undergone cancer surgery >90 days prior to NSCLC diagnosis. Patients with unknown stage of disease were excluded.

In determining utilization of mediastinal biopsy, OHIP billing codes for mediastinoscopy and endobronchial ultrasound (EBUS) were queried and included. For the purposes of determining utilization of CRT, this was defined as having 1 radiation treatment between first and last chemotherapy or 1 chemotherapy treatment between first and last radiation. Radiation dose was required to be at least 40 Gy (to reflect radical radiation in context of CRT), and receipt of chemotherapy was identified through OHIP codes. Use of FDG-PET/CT was determined by OHIP billing codes for FDG-PET/CT scan readings and were categorized as being either prior to diagnosis or post-diagnosis but prior to 1^st^ treatment (either chemotherapy, radiation, CRT, or surgery). Demographic data collected for each patient included: age, sex, geographic location, and income quintile. Oncologic data collected included: date of diagnosis, NSCLC stage, date of FDG-PET/CT scan (if performed), oncologic therapies received (radiotherapy, chemotherapy, chemoradiotherapy, surgery), date of first treatment, Charlson Comorbidity Index, and date of death (if applicable) or last follow-up prior to 31 March 2019.

### 2.3 Analysis plan

As previously discussed, the first objective of this study was to determine the influence of FDG-PET/CT prior to diagnosis or treatment on therapy choice, either chemotherapy alone, radiotherapy alone, CRT or surgery. A subgroup analysis was performed focusing only on the years 2014-2017 when FDG-PET/CT utilization had plateaued and was relatively stable. The second objective was to determine the impact of FDG-PET/CT on survival on the subgroup of stage III NSCLC treated with curative CRT. Secondary objectives included the type and utilization of mediastinal biopsies, timing of FDG-PET/CT relative to initiation of any treatment in the entire cohort, as well as the years 2014-2017 using cut off dates of FDG-PET/CT scans ≤28 days prior to treatment start, 29-56 days prior to treatment, and >56 days prior to treatment. These intervals were chosen based on previous evidence suggesting poorer outcomes with imaging to treatment intervals of >3 weeks (6), where there is an increased probability of up-staging when intervals are ≥24 days (7). Analyses were performed to determine the effect of FDG-PET/CT on survival in a) the entire cohort, b) in patients who received CRT (+/- surgery), and c) in patients who only received CRT.

Descriptive statistics were used to summarize the characteristics and outcomes of all patients included in this analysis, as well as to describe the utilization of FDG PET scans over time. 95%, two-sided, confidence intervals were included for relevant outcomes. Logistic regression analyses were used to evaluate factors prognostic of therapy choice. Overall survival (OS) was calculated from the date of diagnosis until the date of death, or last known contact with the health care system. To account for survival bias, a landmark analysis was performed based on the landmark time of 180 days (6 months), by which time most patients would be expected to have undergone their initial treatment regimen for their primary cancer. The Kaplan-Meier method was used to estimate survival times. Cox proportional hazards regression was performed to explore factors prognostic of OS beyond the landmark time. To explore the impact of FDG-PET/CT, a multivariable model was constructed including all relevant patient characteristics, and receipt of FDG-PET/CT was included adjusting for other factors. With a paucity of missing data (complete data were available for >99% of all individuals in this data set), a complete case analysis was performed. Given the large sample size, traditional statistical significance levels of alpha <0.05 would be met even with small variations from the null hypothesis. No adjustment was performed for multiple testing; however, inferences were performed with caution.

## 3 Results

**Figure 1:**
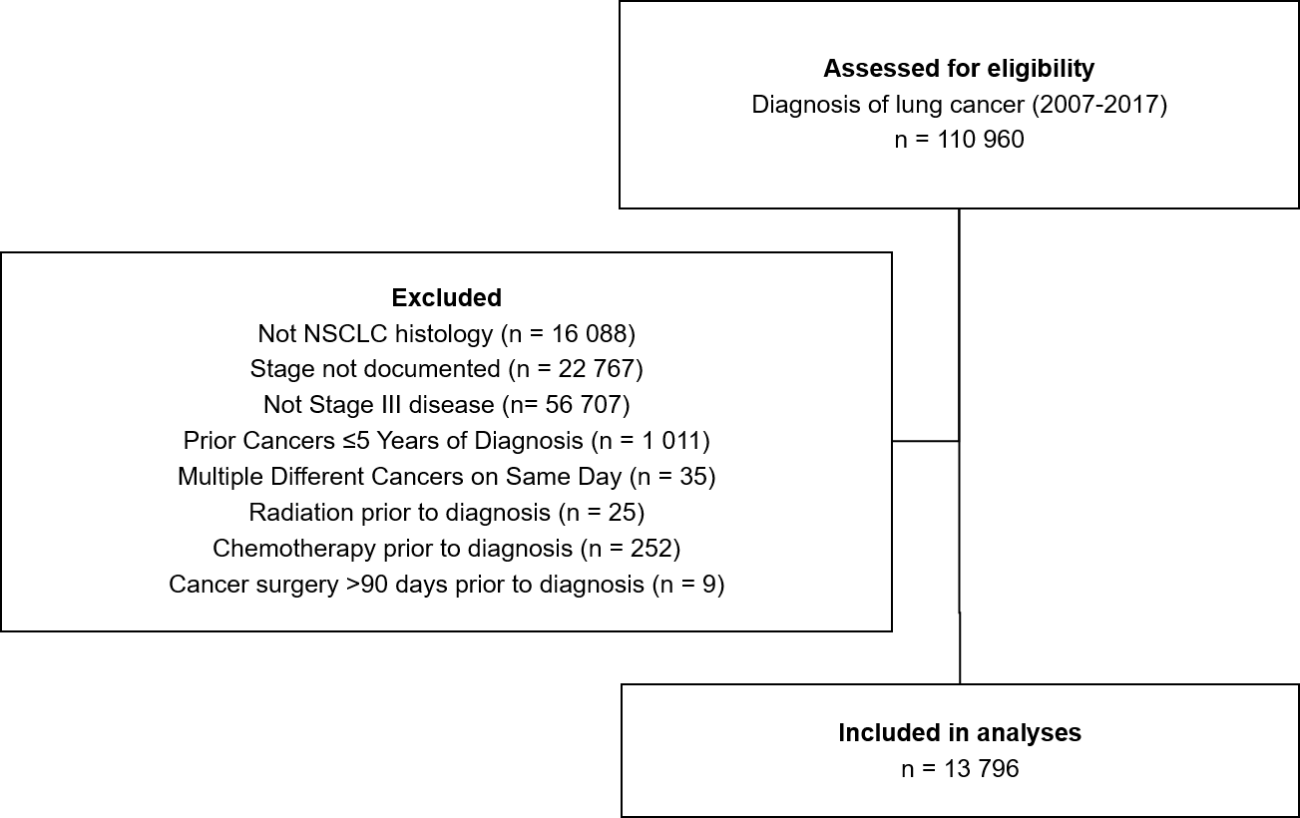
Consolidated standards of reporting trials schema.

Between 2007 and 2017, a total of 13 796 people were diagnosed with stage III NSCLC in Ontario, Canada (**Figure 1**). A mean of 1254 diagnoses were made each year (range 1149-1391, **Table 1**). Males represented the majority of the cases at 54%. The age group with the largest proportion of diagnoses was 70-74 years, having 18% of cases. **Figure 1** depicts the Cohort Identification diagram.

**Table 1:** Subject Demographics.

	N		Total (%)
Year of Diagnosis	13796	2007 2008 2009 2010 2011 2012 2013 2014 2015 2016 2017	1340 (9.7) 1357 (9.8) 1391 (10.1) 1149 (8.3) 1185 (8.6) 1314 (9.5) 1216 (8.8) 1222 (8.9) 1177 (8.5) 1200 (8.7) 1245 (9.0)
Sex	13796	Male	7377 (53.5)
Age Group	13796	<55 55-59 60-64 65-69 70-74 75-79 80-84 85+	1097 (8.0) 1234 (8.9) 1784 (12.9) 2260 (16.4) 2450 (17.8) 2223 (16.1) 1702 (12.3) 1046 (7.6)
Income Quintile	13770	1 2 3 4 5	3421 (24.8) 3136 (22.8) 2627 (19.1) 2470 (17.9) 2116 (15.4)
Rurality	13792	Yes	2243 (16.3)

**Figure 2:**
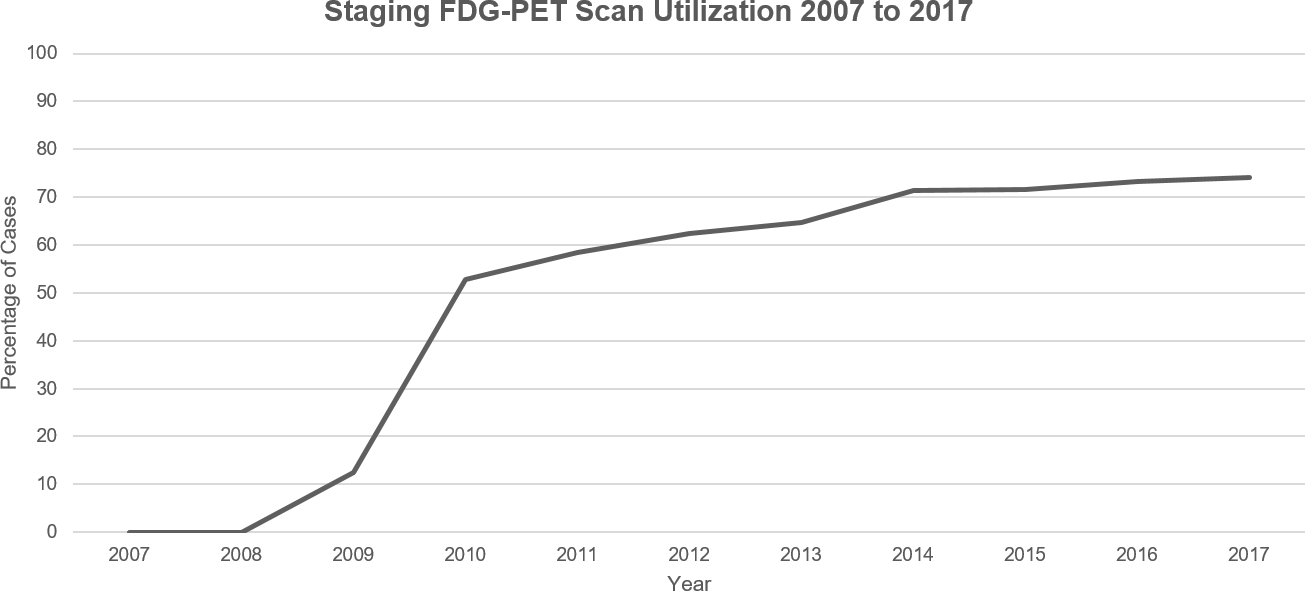
Visualization of the FDG-PET scan utilization trend in Ontario, Canada for new cases of NSCLC between 2007 and 2017.

Pre-treatment FDG-PET/CT utilization steadily increased over the examined time period: 0% utilization was seen in 2007 prior to FDG-PET/CT-staging scans becoming standard of care. By 2017, utilization increased to 74% (**Figure 2**, **Supplementary Table 4**).

### 3.1 Influence of FDG-PET/CT on therapy choice

Of patients who received an FDG-PET/CT scan prior to treatment, 87% received at least one treatment modality (radiotherapy, chemotherapy, chemoradiotherapy, surgery) vs 63% of those who did not receive a pre-treatment FDG-PET/CT scan (p<0.001). Similarly, significantly more patients in the FDG-PET/CT scan group received radiotherapy (65% vs 48%, p<0.001) and chemotherapy (57% vs 35%, p<0.001).

Twenty-three percent of patients who received a pre-treatment FDG-PET/CT scan received CRT compared with 13% of people who did not receive an FDG-PET/CT scan (p<0.001). Findings were similar when looking specifically at surgical intervention, as 23% of patients scanned with FDG-PET/CT received surgical intervention, while only 10% of people without PET scans underwent surgery (p<0.001). Further data regarding the effect of PET of treatment choice can be found in **Supplementary Table 5**.

### 3.2 Trends in biopsy usage

**Figure 3:**
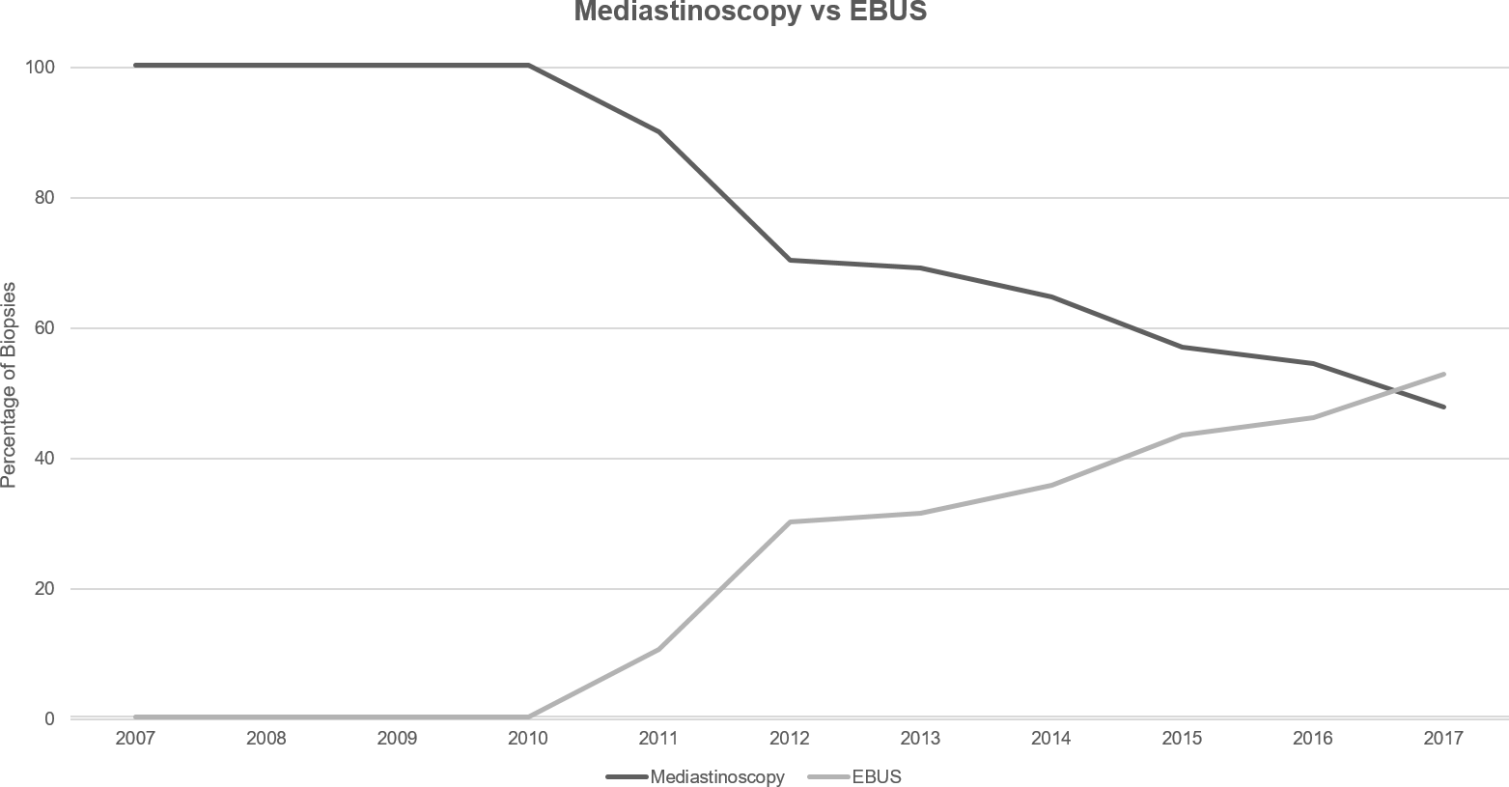
Technique for mediastinal staging between 2007 and 2017.

Utilization of any mediastinal staging increased over the analyzed timeframe from 41% of the annual cohort of Stage III NSCLC patients in 2007 to 53% in 2017 (**Supplementary Table 3**). Subdividing mediastinal biopsy technique between surgical mediastinoscopy and EBUS, usage of mediastinoscopy decreased between 2007 and 2017 from 100% of biopsies to 47% (**Figure 3**). Conversely, EBUS utilization rose from 0% to 53% in 2017.

### 3.3 Influence of FDG-PET/CT on survival

**Figure 4:**
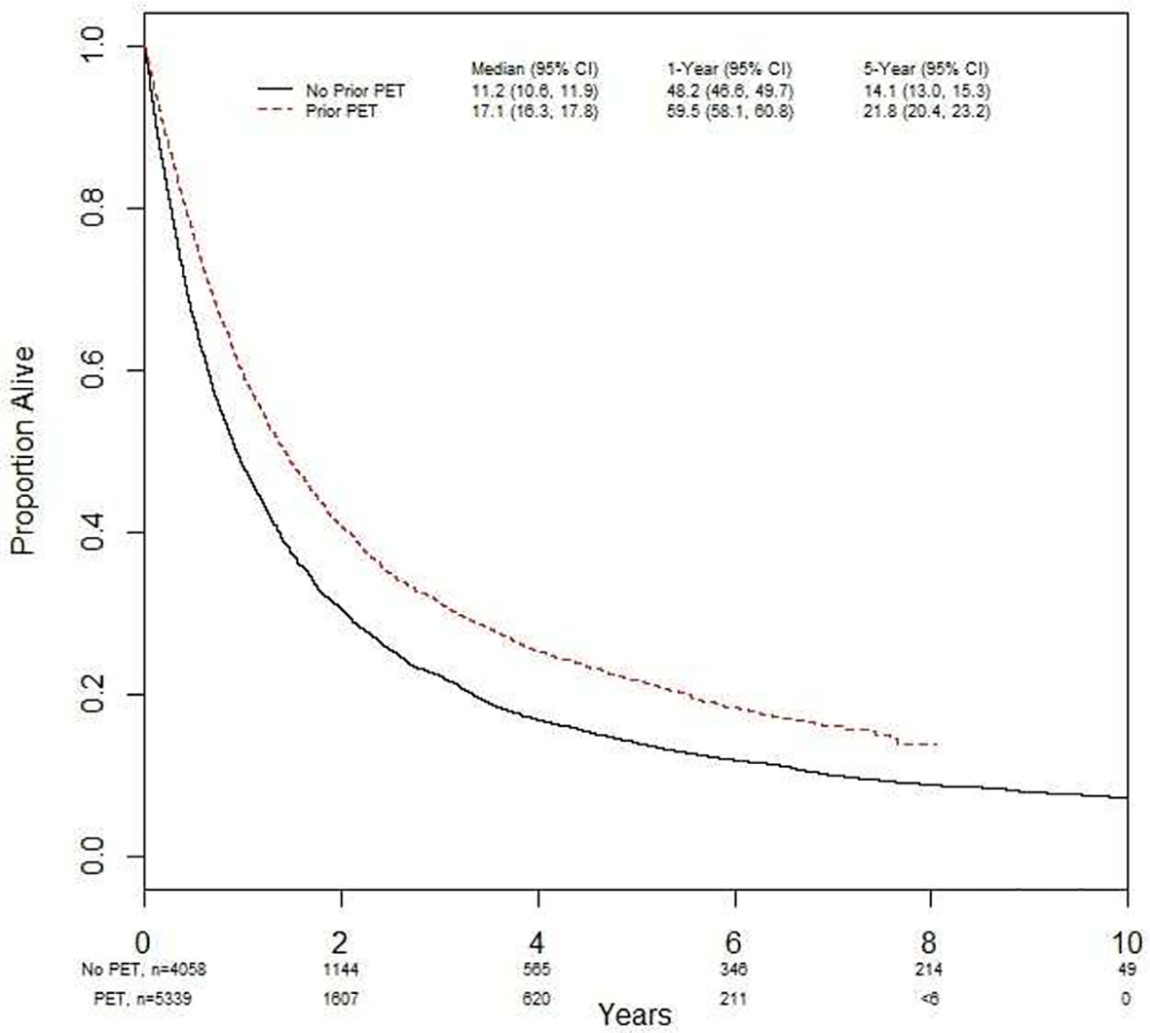
Impact of FDG-PET scans prior to treatment on overall survival.

Using a landmark analysis of 6 months, OS was investigated in the entire population studied (**Figure 4**). In the time period examined, 66% (n=3514) of the people from FDG-PET/CT scan cohort died versus 86% (n=3507) of those without an FDG-PET/CT scan. The median OS with an FDG-PET/CT scan was 17.0 months (95% CI 16.3, 17.8), and 11 months (95% CI 10.6, 11.9) without (p<0.001). The 5-year survival was 22% (95% CI 20.4, 23.2) with FDG-PET/CT scan, and 14% (95% CI 13.0, 15.3) without (p<0.001). Undergoing FDG-PET/CT scan prior to treatment was significantly associated with overall survival in both univariate (HR = 0.75 [95% CI = 0.71, 0.78]) and multivariate analyses (HR = 0.80 [95% CI = 0.76, 0.85]) (**Table 2**).

**Table 2:** Prognostic factors for overall survival in entire cohort.

Univariate analysis			
Prognostic factor	Variable	HR (95% CI)	p-value
Sex	Male vs Female	1.20 (1.15, 1.26)	<0.001
Known Charlson Score	>=1 vs 0	1.29 (1.20, 1.37)	<0.001
Radiotherapy alone	Yes vs No	1.20 (1.14, 1.26)	<0.001
Chemoradiotherapy	Yes vs No	0.63 (0.60, 0.66)	<0.001
Surgery	Yes vs No	0.43 (0.40, 0.46)	<0.001
PET Prior to Treatment	Yes vs No	0.75 (0.71, 0.78)	<0.001
Year of Diagnosis	Year	0.99 (0.98, 0.99)	<0.001
Income Quintile	Quintile	0.96 (0.95, 0.98)	<0.001
Rurality	Yes vs No	1.05 (0.99, 1.12)	0.13

Multivariate analysis			
Prognostic factor	Variable	HR (95% CI)	p-value
Sex	Male vs Female	1.18 (1.13, 1.24)	<0.001
Known Charlson Score	>=1 vs 0	1.05 (0.98, 1.13)	0.15
Chemoradiotherapy	Yes vs No	0.69 (0.65, 0.74)	<0.001
Surgery	Yes vs No	0.43 (0.40, 0.46)	<0.001
PET Prior to Treatment	Yes vs No	0.80 (0.76, 0.85)	<0.001
Year of Diagnosis	Year	1.00 (0.99, 1.01)	0.75
Income Quintile	Quintile	0.97 (0.95, 0.99)	<0.001
Rurality	Yes vs No	1.04 (0.97, 0.99)	0.26

In the subgroup of stage III NSCLC treated with curative CRT, having an FDG-PET/CT scan prior to treatment was associated with improved OS in both univariate (HR 0.86 [95% CI 0.78, 0.95], p = 0.004) and multivariate (HR 0.83 [95% CI 0.72, 0.95], p=0.006) analyses (**Supplementary Table 1**). Patient sex and surgery were also found to significantly impact the hazard ratio in both univariate and multivariate analyses (**Supplementary Table 1**). Results were similar in patients who were treated with curative CRT +/- surgery (**Supplementary Table 2**).

### 3.4 Timing of FDG-PET/CT relative to OS

**Table 3** shows the timing of when patients received an FDG-PET/CT prior to treatment and their subsequent OS. Median OS was 16 months (95% CI 14.5, 17.7) when FDG-PET/CT scan was performed ≤28 days prior to treatment, 18 months (95% CI 16.3, 19.6) if between 29-56 days prior to treatment, and 19 months (95% CI 17.0, 20.9) when >56 days prior to treatment. Differences in median OS were not significantly different between groups (p = 0.38). Looking specifically from years 2014-2017, the OS numbers were similar in this subgroup.

**Table 3:** Timing of PET scan and influence on overall survival.

		PET ≤28 Days Prior to Treatment	PET 29 to 56 Days Prior to Treatment	PET >56 Days Prior to Treatment	p-value
Entire cohort					
**N**		vv1842	2149	1482	n/a
**Median Days between Dx and** **Treatment (IQR)**		42 (30, 56)	55 (40, 74)	88 (63, 112)	n/a
**Overall Survival (Landmark of 6** **months)**	N Median Months 95% CI	1481 16 14.5, 17.7	1829 17.8 16.3, 19.6	1311 18.6 17.0, 20.9	0.38
2014-2017 only					
**N**		1842	2149	1482	n/a
**Median Days between Dx and** **Treatment (IQR)**		40 (28, 54)	52 (37, 68)	83 (60, 106)	n/a
**Overall Survival (Landmark of 6** **months)**	N Median Months 95% CI	721 15.2 12.9, 17.5	991 17.2 15.3, 19.4	676 18.3 16.1, 24.2	0.091

## 4 Discussion

This report provides a retrospective look at the impact of pre-treatment FDG-PET/CT scans for staging of new cases of NSCLC in Ontario, Canada from 2007 to 2017. During that timeframe, utilization of pre-treatment FDG-PET/CT increased from 0% in 2007 to 74% in 2017. Mediastinal biopsy utilization increased from 41% to 53% in 2017, while mediastinoscopy usage decreased as more patients received EBUS biopsy. More stage III NSCLC patients who had FDG-PET/CT scans received at least one oncologic treatment, and received curative-intent treatment such as CRT +/- surgery. Overall survival for subjects undergoing an FDG-PET/CT scan was greater than those without. However, no observed relationship was seen between the timing of PET scan and OS.

Published data regarding population utilization of pre-treatment FDG-PET/CT in NSCLC are limited. A large, retrospective cohort study of 64 103 veterans treated within the United States Veteran Affairs (VA) health care system from 2000 to 2013 found that 40.1% (25 735) of the study population underwent an FDG-PET/CT in the year before diagnosis, while 64.3% (41 242) were scanned in the 5 years after diagnosis (8). Though the authors do not explicitly delineate pre-treatment FDG-PET/CT, nor show trends in utilization, they do show that approximately 75% of patients received FDG-PET/CT within 3 months of their diagnosis. This is quite similar to data presented herein which showed 74% FDG-PET/CT utilization for stage III NSCLC cases in Ontario for 2017. Another study from the United States using the Surveillance, Epidemiology, and End Results (SEER)-Medicare linked database reported that for new cases of NSCLC, 78% of non-Hispanic white people received FDG-PET/CT between 3 months before diagnosis and up to 30 days after initiation of therapy (9). The authors also found that 63% of black people and 70% of Hispanic people received FDG-PET/CT scans following the above criteria and argue that this disparity may explain differences in survival statistics between these ethnic groups. The cohort of patients reported in this project were not stratified by race; given the findings from the SEER database, this is an avenue for further research that should be pursued.

It is well established in the literature that pre-treatment FDG-PET/CT has a meaningful impact on treatment choice for new cases of NSCLC: work from 2001 found that the inclusion of FDG-PET/CT scans to staging investigations often demonstrated previously undetected metastases leading to appropriate staging and strongly influencing treatment strategy (10). Further work from 2001 on 105 cases of NSCLC where FDG-PET/CT was used for primary staging or re-staging showed that in 26% of the cases, results of the FDG-PET/CT scan led to a change in management plan from curative to palliative intent therapy (11). Indeed, as this was a prospective study, the authors determined that findings from the FDG-PET/CT scans changed or influenced management decisions in 67% of cases. The authors argue that these changes helped spare unnecessary treatment and ensure disease-appropriate management plans. More recent work from 2009 utilized questionnaire data from treating physicians to examine changes to treatment plans before and after FDG-PET/CT scans across all oncologic diagnoses, including lung cancer (12). Though not limited to NSCLC, the authors present data from 8240 cases and showed meaningful changes in planned treatment: 26.5% of cases where changes to type of therapy were made, 16.6% where changes were made to dose or duration of therapy, and 6.2% of subjects where the decision was made to change from therapy to observation or supportive care. Two more recent studies from 2020 point to the ability of FDG-PET/CT scans to aid in treatment choice of certain newly approved therapies including tyrosine kinase inhibitors (TKI) and immune checkpoint inhibitors (ICI): there was good performance in predicting epidermal growth factor receptor (EGFR) mutation status in NSCLC cases, potentially providing a faster alternative method for EGFR target therapy choice (13, 14).

The reasoning behind the observed increase in curative-intent therapies in patients who received a staging PET scan is challenging to explain and is likely multifactorial. One component may be related to provider comfort level in treating patients more aggressively that have been staged with PET-CT. Indeed, work examining radiation target volume delineation in patients with and without staging PET scans, showed reduced interobserver variability with fused PET-CT scans (15) and higher proportions of patients with PET scans receiving radical radiation doses (16). A second factor which may impact curative-intent treatment decision making is performance status: it is well established that pre-treatment performance status is a strong predictor of patient outcome with curative-intent NSCLC therapies (17), therefore patients with poorer performance status may be less likely to receive curative-intent therapy, and may also be less likely to receive staging PET-CT scans. Additionally, unequal patient access to PET scanners may represent a third contributing factor to the increase in curative-intent therapies in patients who received a staging PET scan. As northern parts of Ontario have poorer access to advanced scanning modalities such as PET-CT (18), this inequality may lead to fewer patients receiving curative-intent therapies, and ultimately having worse outcomes.

Looking at the trends for mediastinal staging, utilization of any mediastinal biopsy showed an increase over the timeframe examined, though not as dramatic as the increased PET utilization previously discussed. A major component of this trend likely represents clinicians seeking histopathological diagnosis for PET-avid lymph nodes. Targeting FDG-PET/CT positive lymph nodes with mediastinal biopsy has demonstrated reduced false-negative and false positive rates (19) and provides more accurate staging information than either modality alone (20). The observed trend of increasing EBUS usage when compared to mediastinoscopy for histopathological diagnosis is not a surprising finding given the less-invasive nature, lack of requirement for general anesthesia, and the lower complication rate of EBUS biopsies (21–23). The role that FDG-PET/CT may play in the increased adoption of EBUS is less clear. As previously discussed, EBUS biopsies guided by FDG-PET/CT data has been shown to have higher diagnostic accuracy than either technique alone (19). However, as PET-CT has now become standard-of-care for investigation and staging in NSCLC per ESMO guidelines, precise determination of the cause for the trend observed in the data presented here is unlikely.

The observed improved survival amongst patients undergoing FDG-PET/CT imaging is likely related to improved staging and the resulting pursuit of more aggressive therapies based on FDG-PET/CT results, rather than a therapeutic consequence of an FDG-PET/CT scan. While treatment decisions may play some role in improved survival, the impact related to appropriate staging must also be considered. Though FDG-PET/CT scans do not have a definitive role in diagnosing malignant pleural effusions currently (24), the remaining indications for M1 staging in NSCLC can be reliably detected and are key determining factors for appropriate staging. A large cohort study published in 2019 demonstrated that even though appropriate initial staging with FDG-PET/CT scans does account for stage-specific improvements in OS, the more impactful effect of FDG-PET/CT scans is through providing stage-appropriate therapies (8). In this report, the authors cite significant improvement in mortality across all NSCLC stages with the addition of FDG-PET/CT scans as their reason for the hypothesis that the association between accurate staging and decreased mortality is related to stage-appropriate therapy rather than related to appropriate staging pre-treatment. As the data presented herein is limited to stage III cases, the effect on OS by appropriate pre-treatment staging and or treatment choice cannot be determined, however it provides an exciting avenue for further investigation.

There was no significant difference seen in OS based on time comparing scans performed ≤28 days, 29-56 days, or >56 days prior to treatment. Though previous work from 2009 established that a shorter time to treatment is associated with improved OS in stage III NSCLC (25), literature examining the impact of delayed FDG-PET/CT scans is quite limited: a prospective trial from 2013 enrolled 82 people diagnosed with NSCLC who received two FDG-PET/CT scans between 8 and 176 days apart as part of their definitive chemoradiotherapy treatment (26). Among their findings, the authors found that a longer time gap between FDG-PET/CT and treatment led to larger radiation fields and increased tumour volume. An analysis of this kind would be beyond the scope of the project presented herein due to the number of subjects and inability to capture all radiation plans retrospectively. Diagnostic and treatment delays are expected to cause a substantial increase in the number of avoidable deaths due to cancer in the near future (27). Hopefully, the data presented in this project which demonstrated no significant relationship between delayed FDG-PET/CT scans and OS from 2007 to 2017 can be reassuring especially as delays from diagnosis to treatment are still present, and have been highlighted further during the COVID-19 pandemic.

The work presented here has limitations. First, during the timeframe studied (2007 to 2017), the AJCC Cancer Staging Manual went through three iterations (28–30): the 6^th^ edition (2002), the 7^th^ edition (2010), and the 8^th^ edition (2017). As each case was staged at the time of diagnosis, the updates between editions may have led to some variability between stage classifications. Secondly, the data analyzed was limited to stage III disease. This was an intentional decision by the authors to answer the research questions posed, however, this may limit the applicability of the results outside of stage III disease. Lastly, the geography and population distribution on Ontario can lead to challenges in providing equal access to cancer care across the province (18). As per Statistics Canada, Ontario has a land area of greater than 908 000 km^2^ and a population over 14 million (2020) that is served by 13 FDG-PET/CT centres in 7 cities (Cancer Care Ontario, https://www.ccohealth.ca/en/). While the issue of unequal access to FDG-PET/CT scans across Ontario does not explicitly alter the data analyses presented herein, it speaks to a larger issue of diagnostic access in rural areas of Canada.

## 5 Conclusions

This project provided a look at the implementation of FDG-PET/CT scans as standard of care for NSCLC staging in Ontario, Canada over 10 years. Based on these data, the addition of pre-treatment FDG-PET/CT scans led to improved overall survival for patients with stage III NSCLC. This is likely due to improved stage-appropriate therapy and reduced stage misclassification, emphasizing the importance of pre-treatment FDG-PET/CT scans. Further and advocacy needs to be performed to reduce gaps in access, and to ensure that all patients who are eligible for curative-intent therapy receive an FDG-PET/CT scan as part of their initial work-up for suspected stage III NSCLC.

## Data availability statement

The original contributions presented in the study are included in the article/**Supplementary Material**, further inquiries can be directed to the corresponding author.

## Ethics statement

Study ethics approval was obtained through the local institutional Hamilton Integrated Research Ethics Board and the Data Analytics Systems programs through IC/ES.

## Author contributions

All co-authors contributed to conception and design of the study. GP organized the database. AS, CB & GP performed the statistical analysis. CB wrote the first draft of the manuscript. All authors contributed to manuscript revision, read, and approved the submitted version.
