# Supplementary material for: The impact of staging FDG-PET/CT on treatment for stage III NSCLC - an analysis of population-based data from Ontario, Canada
Source: Front Oncol. 2023 Aug 23;13:1210945. doi: 10.3389/fonc.2023.1210945 (PMC10482027; doi:10.3389/fonc.2023.1210945)
Supplement: Supplementary file 1 [file Presentation_1.pptx]

## Slide 1
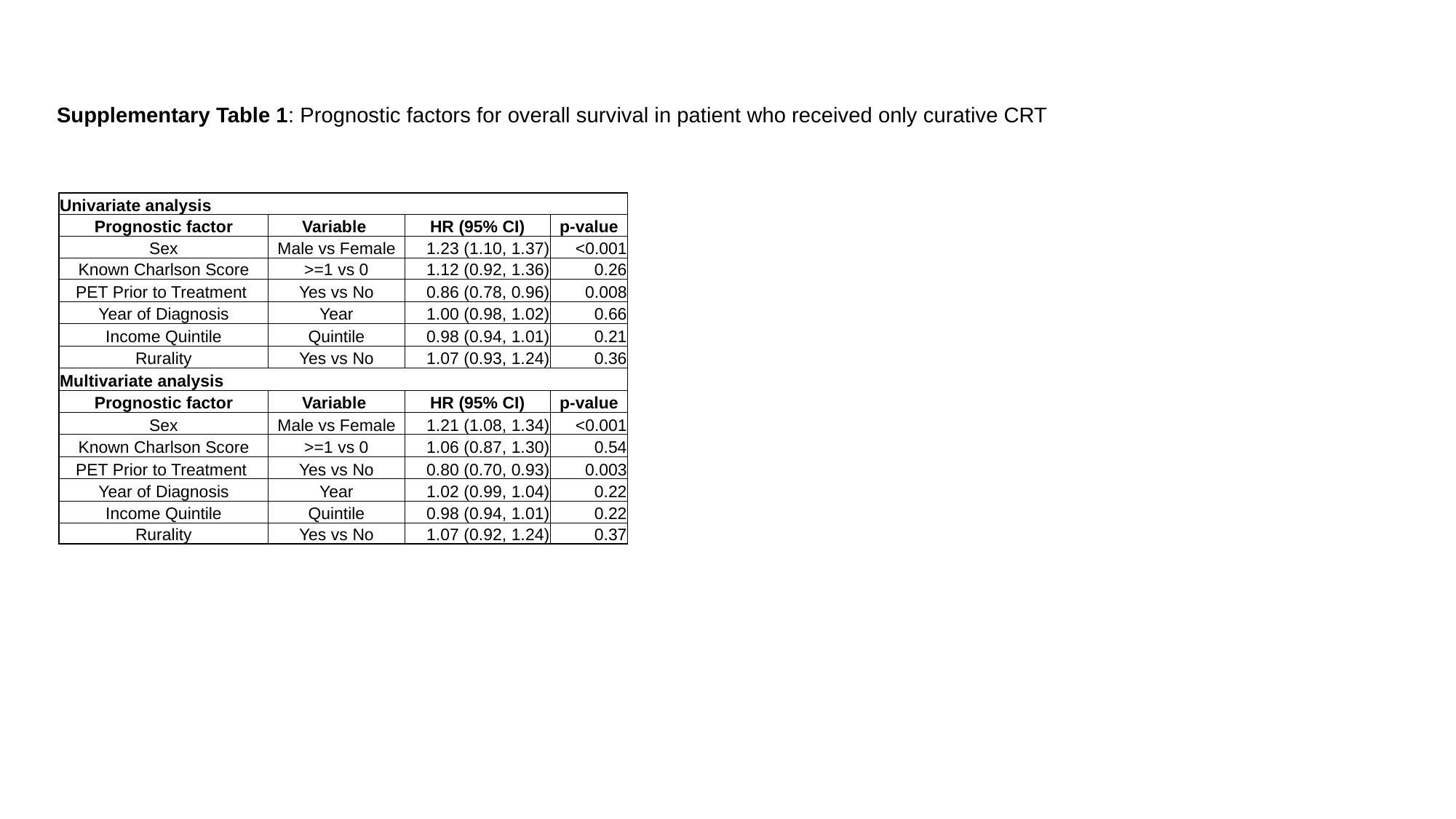

Supplementary Table 1: Prognostic factors for overall survival in patient who received only curative CRT
| Univariate analysis | | | |
| --- | --- | --- | --- |
| Prognostic factor | Variable | HR (95% CI) | p-value |
| Sex | Male vs Female | 1.23 (1.10, 1.37) | <0.001 |
| Known Charlson Score | >=1 vs 0 | 1.12 (0.92, 1.36) | 0.26 |
| PET Prior to Treatment | Yes vs No | 0.86 (0.78, 0.96) | 0.008 |
| Year of Diagnosis | Year | 1.00 (0.98, 1.02) | 0.66 |
| Income Quintile | Quintile | 0.98 (0.94, 1.01) | 0.21 |
| Rurality | Yes vs No | 1.07 (0.93, 1.24) | 0.36 |
| Multivariate analysis | | | |
| Prognostic factor | Variable | HR (95% CI) | p-value |
| Sex | Male vs Female | 1.21 (1.08, 1.34) | <0.001 |
| Known Charlson Score | >=1 vs 0 | 1.06 (0.87, 1.30) | 0.54 |
| PET Prior to Treatment | Yes vs No | 0.80 (0.70, 0.93) | 0.003 |
| Year of Diagnosis | Year | 1.02 (0.99, 1.04) | 0.22 |
| Income Quintile | Quintile | 0.98 (0.94, 1.01) | 0.22 |
| Rurality | Yes vs No | 1.07 (0.92, 1.24) | 0.37 |

## Slide 2
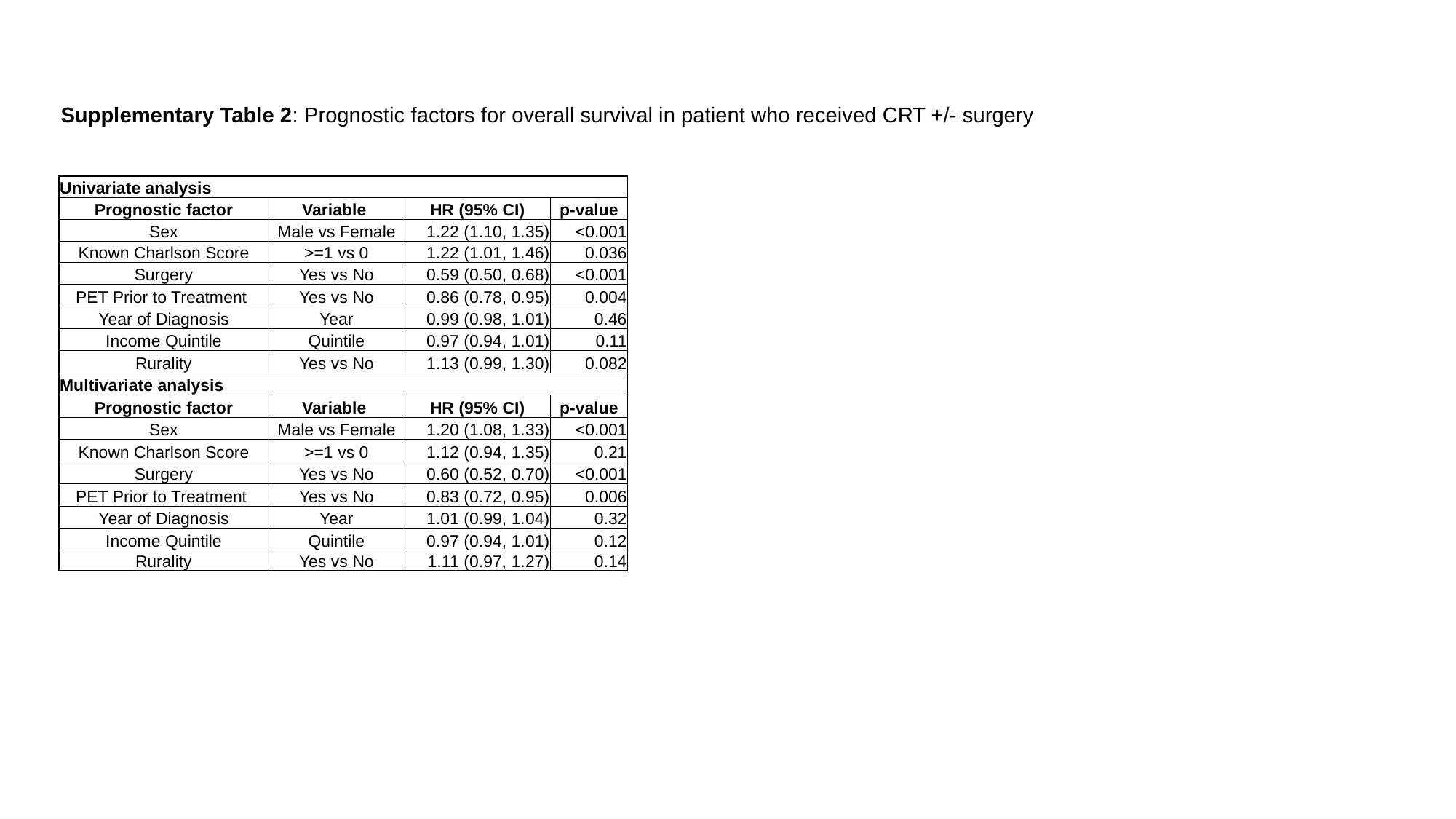

Supplementary Table 2: Prognostic factors for overall survival in patient who received CRT +/- surgery
| Univariate analysis | | | |
| --- | --- | --- | --- |
| Prognostic factor | Variable | HR (95% CI) | p-value |
| Sex | Male vs Female | 1.22 (1.10, 1.35) | <0.001 |
| Known Charlson Score | >=1 vs 0 | 1.22 (1.01, 1.46) | 0.036 |
| Surgery | Yes vs No | 0.59 (0.50, 0.68) | <0.001 |
| PET Prior to Treatment | Yes vs No | 0.86 (0.78, 0.95) | 0.004 |
| Year of Diagnosis | Year | 0.99 (0.98, 1.01) | 0.46 |
| Income Quintile | Quintile | 0.97 (0.94, 1.01) | 0.11 |
| Rurality | Yes vs No | 1.13 (0.99, 1.30) | 0.082 |
| Multivariate analysis | | | |
| Prognostic factor | Variable | HR (95% CI) | p-value |
| Sex | Male vs Female | 1.20 (1.08, 1.33) | <0.001 |
| Known Charlson Score | >=1 vs 0 | 1.12 (0.94, 1.35) | 0.21 |
| Surgery | Yes vs No | 0.60 (0.52, 0.70) | <0.001 |
| PET Prior to Treatment | Yes vs No | 0.83 (0.72, 0.95) | 0.006 |
| Year of Diagnosis | Year | 1.01 (0.99, 1.04) | 0.32 |
| Income Quintile | Quintile | 0.97 (0.94, 1.01) | 0.12 |
| Rurality | Yes vs No | 1.11 (0.97, 1.27) | 0.14 |

## Slide 3
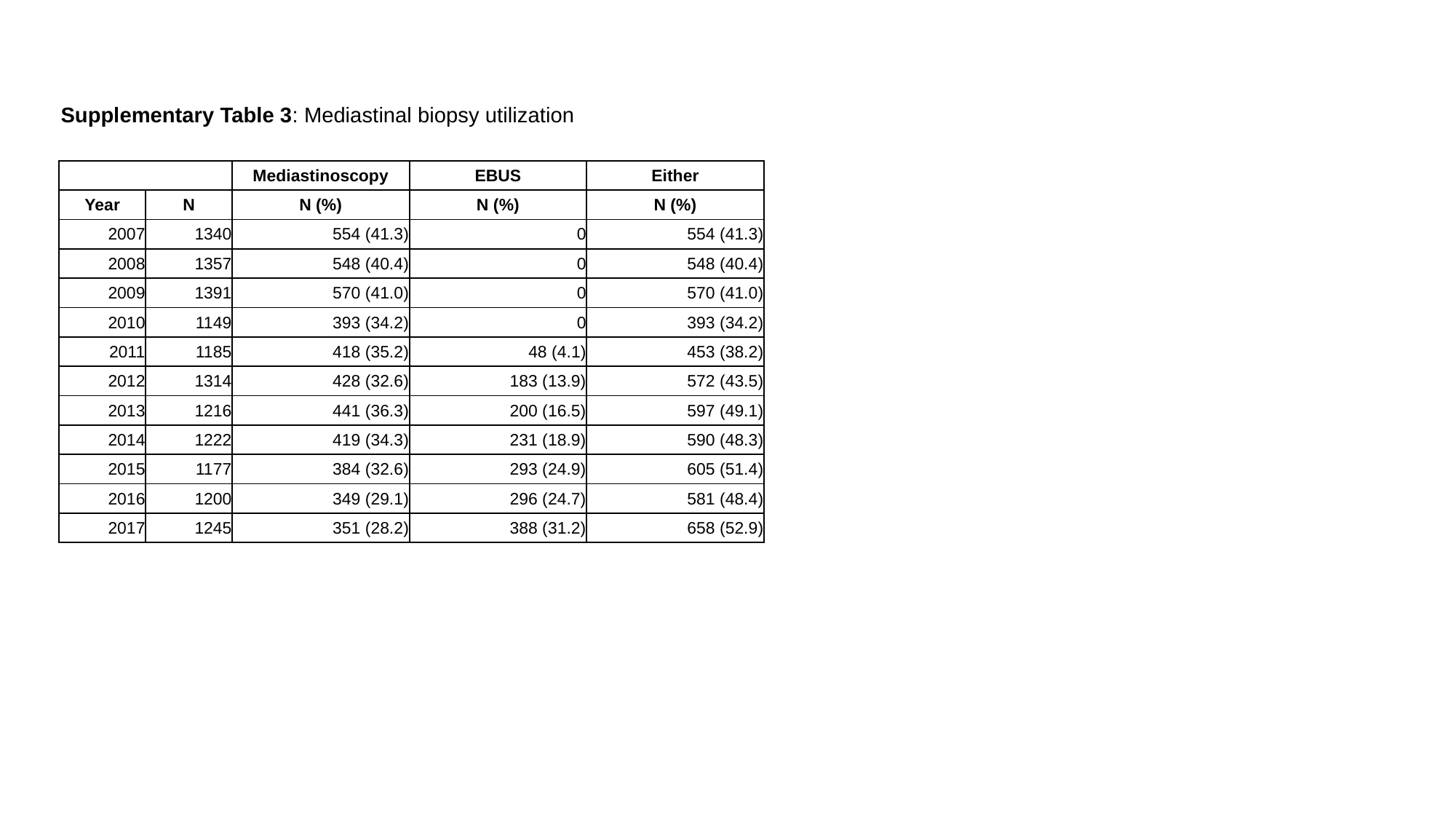

Supplementary Table 3: Mediastinal biopsy utilization
| | | Mediastinoscopy | EBUS | Either |
| --- | --- | --- | --- | --- |
| Year | N | N (%) | N (%) | N (%) |
| 2007 | 1340 | 554 (41.3) | 0 | 554 (41.3) |
| 2008 | 1357 | 548 (40.4) | 0 | 548 (40.4) |
| 2009 | 1391 | 570 (41.0) | 0 | 570 (41.0) |
| 2010 | 1149 | 393 (34.2) | 0 | 393 (34.2) |
| 2011 | 1185 | 418 (35.2) | 48 (4.1) | 453 (38.2) |
| 2012 | 1314 | 428 (32.6) | 183 (13.9) | 572 (43.5) |
| 2013 | 1216 | 441 (36.3) | 200 (16.5) | 597 (49.1) |
| 2014 | 1222 | 419 (34.3) | 231 (18.9) | 590 (48.3) |
| 2015 | 1177 | 384 (32.6) | 293 (24.9) | 605 (51.4) |
| 2016 | 1200 | 349 (29.1) | 296 (24.7) | 581 (48.4) |
| 2017 | 1245 | 351 (28.2) | 388 (31.2) | 658 (52.9) |

## Slide 4
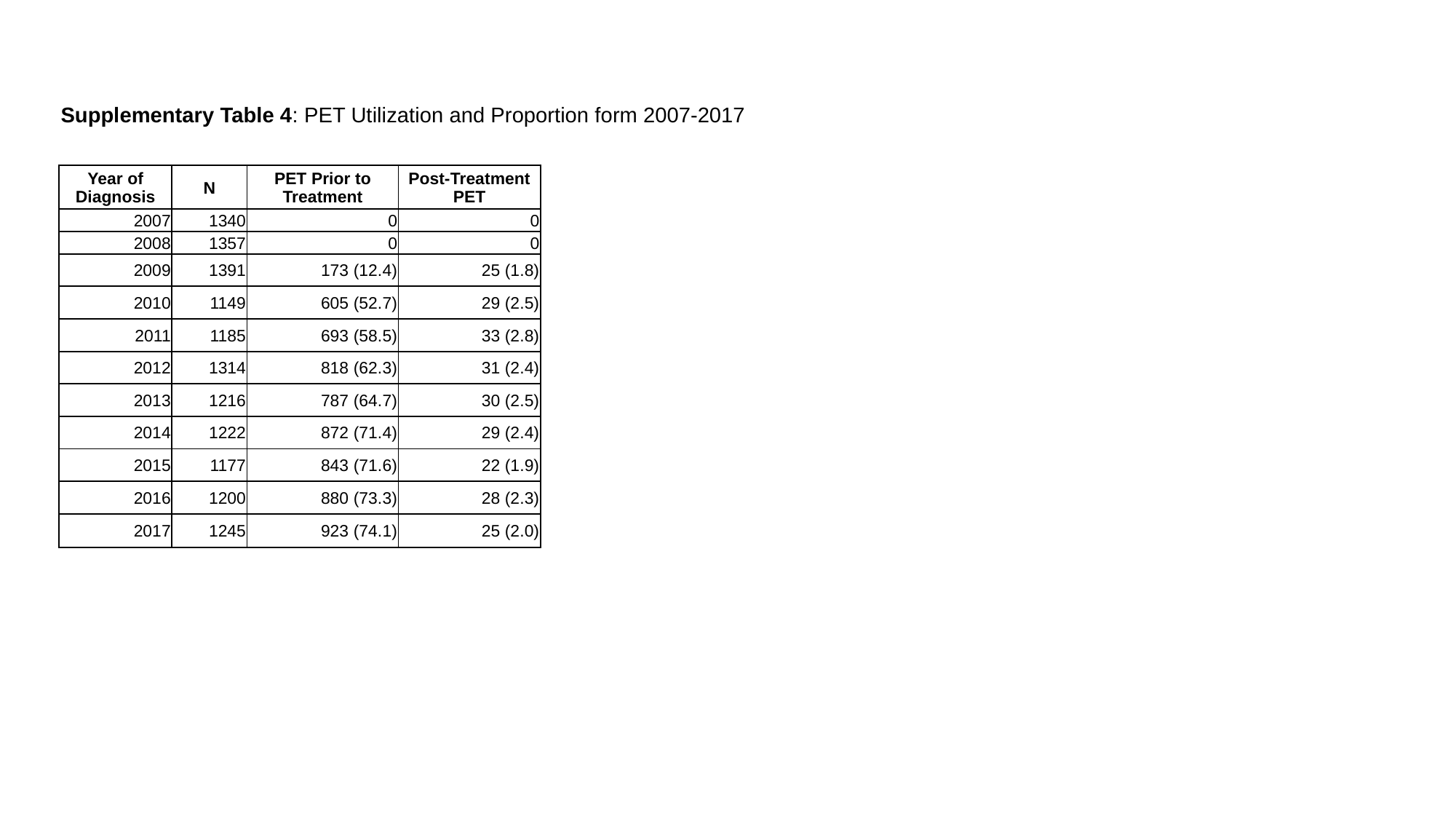

Supplementary Table 4: PET Utilization and Proportion form 2007-2017
| Year of Diagnosis | N | PET Prior to Treatment | Post-Treatment PET |
| --- | --- | --- | --- |
| 2007 | 1340 | 0 | 0 |
| 2008 | 1357 | 0 | 0 |
| 2009 | 1391 | 173 (12.4) | 25 (1.8) |
| 2010 | 1149 | 605 (52.7) | 29 (2.5) |
| 2011 | 1185 | 693 (58.5) | 33 (2.8) |
| 2012 | 1314 | 818 (62.3) | 31 (2.4) |
| 2013 | 1216 | 787 (64.7) | 30 (2.5) |
| 2014 | 1222 | 872 (71.4) | 29 (2.4) |
| 2015 | 1177 | 843 (71.6) | 22 (1.9) |
| 2016 | 1200 | 880 (73.3) | 28 (2.3) |
| 2017 | 1245 | 923 (74.1) | 25 (2.0) |

## Slide 5
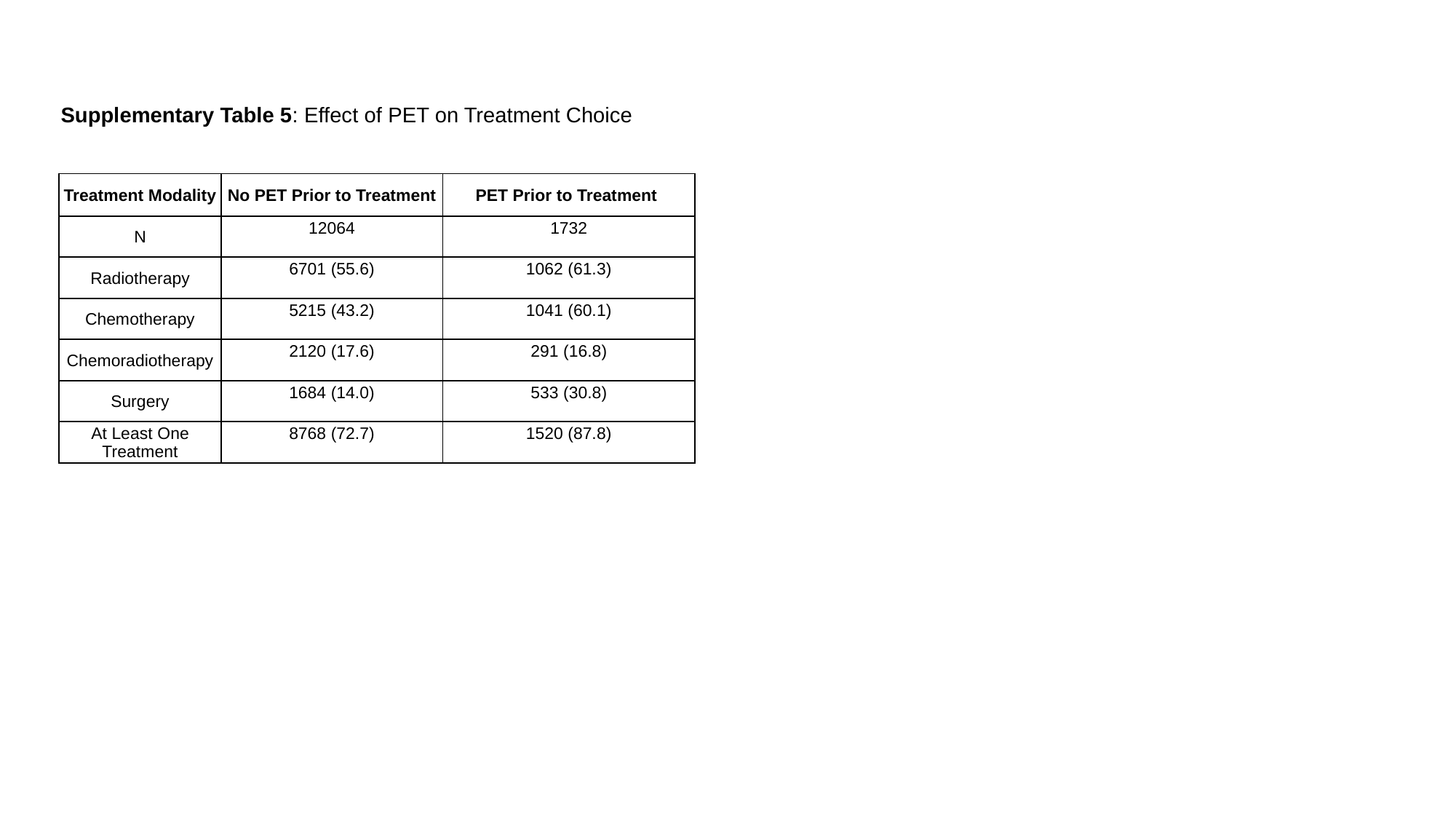

Supplementary Table 5: Effect of PET on Treatment Choice
| Treatment Modality | No PET Prior to Treatment | PET Prior to Treatment |
| --- | --- | --- |
| N | 12064 | 1732 |
| Radiotherapy | 6701 (55.6) | 1062 (61.3) |
| Chemotherapy | 5215 (43.2) | 1041 (60.1) |
| Chemoradiotherapy | 2120 (17.6) | 291 (16.8) |
| Surgery | 1684 (14.0) | 533 (30.8) |
| At Least One Treatment | 8768 (72.7) | 1520 (87.8) |
